# Social Determinants of Health and Patients’ Technology Acceptance of Telehealth During the COVID-19 Pandemic: Pilot Survey

**DOI:** 10.2196/47982

**Published:** 2023-11-07

**Authors:** Sneha Anil Kumar Vaidhyam, Kuo-Ting Huang

**Affiliations:** 1 Department of Information Culture and Data Stewardship School of Computing and Information University of Pittsburgh Pittsburgh, PA United States

**Keywords:** social determinants of health, telehealth, COVID-19, technology adoption

## Abstract

**Background:**

Telehealth has been widely adopted by patients during the COVID-19 pandemic. Many social determinants of health influence the adoption.

**Objective:**

This pilot study aimed to understand the social determinants of patients’ adoption of telehealth in the context of the pandemic.

**Methods:**

A survey methodology was used to capture data from 215 participants using Amazon Mechanical Turk. The study was guided by the technology acceptance model and the social determinants of health framework. The questionnaire included technology acceptance model variables (eg, perceived usefulness [PU] and perceived ease of use [PEOU]), social determinants (eg, access to health care, socioeconomic status, education, and health literacy), and demographic information (eg, age, sex, race, and ethnicity). A series of ordinary least squares regressions were conducted to analyze the data using SPSS Statistics (IBM Corp).

**Results:**

The results showed that social determinant factors—safe neighborhood and built environment (*P*=.01) and economic stability (*P*=.05)—are predictors of the PEOU of telehealth adoption at a statistically significant or marginally statistically significant level. Furthermore, a moderated mediation model (PROCESS model 85) was used to analyze the effects of COVID-19 on the neighborhood, built environment, and economic stability. PEOU and PU significantly positively affected users’ intention to use technology for both variables.

**Conclusions:**

This study draws attention to 2 research frameworks that address unequal access to health technologies. It also adds empirical evidence to telehealth research on the adoption of patient technology. Finally, regarding practical implications, this study will provide government agencies, health care organizations, and health care companies with a better perspective of patients’ digital health use. This will further guide them in designing better technology by considering factors such as social determinants of health.

## Introduction

### Background

The COVID-19 pandemic has become a global health emergency, leading to several catastrophic events, leaving thousands dead, millions susceptible, economies disrupted, factories shunted, and cities under lockdown [1,2]. Although the crisis presented the US health care delivery system with unprecedented challenges, it also catalyzed the rapid adoption of digital health tools [3]. Health care organizations rapidly adopted alternative modes of health care delivery, such as telehealth, to help minimize the spread of COVID-19 [4]. Telehealth is considered an effective alternative for providing health care services without the need for close contact and the risk of exposure for patients and clinicians [5,6]. Furthermore, these technologies can potentially increase real-time data sharing and collaboration between health care providers and patients [7].

Telehealth is being leveraged with enormous speed and scale, turning into the forward *front line* of the battle against the pandemic. The emerging literature on the role of telehealth in response to COVID-19 has focused on the health informatics infrastructure and primary care visits [8-10]. However, some barriers prevent telehealth from being widely adopted; these include limited reimbursement, lack of financial stability, lack of education on how to access health care information through the internet, and lack of comfort with telehealth technologies (video chat or webcam and mobile phone) [9,11,12]. Previous literature highlights that patients from underserved populations are mostly affected by these barriers [11,13]. Social determinant factors, such as socioeconomic determinants, education level, insurance status, access to technology, and race impact the acceptance and adoption of health technologies [7,14-16]. Although research on health care systems has been actively exploring social determinants in clinical settings, there is limited research on how these determinants may impact patients’ acceptance of telehealth.

Adopting information technologies has immediate and long-term advantages such as improved productivity, streamlined processes, cost-effectiveness, time efficiency, and improved communication [17]. These benefits of technology adoption have motivated researchers to learn more about the acceptance of innovative technologies by people from various backgrounds. However, only a limited number of studies have explored acceptance of telehealth technology. Numerous conceptual frameworks have been proposed to evaluate acceptance and behaviors related to the adoption of technology [18]. The most renowned among these is the technology acceptance model (TAM), introduced in 1989 [18,19]. Over the years, it has been widely applied and tested across a diverse range of information and communication technologies, including health care. TAM is one of the most widely used research frameworks to predict an individual’s intention to use (IU) technology, assess a particular behavior, and assess overall acceptance [20].

Guided by the social determinants of the health framework and TAM, this study aims to investigate how social determinants predict patients’ adoption of telehealth in the context of the COVID-19 pandemic. Specifically, this study seeks to answer the following questions: (1) do social determinants of health (SDOH) predict patients’ acceptance of telehealth? If so, (2) how do different social factors lead to barriers to the adoption of telehealth? and (3) does being infected with COVID-19 facilitate the acceptance of telehealth? This study intends to highlight areas within this field that may need assessment, improvement, and complete development and, in turn, improve standards and quality of patient care.

### Literature Review

This study uses the TAM to assess how SDOH influence the acceptance and adoption of telehealth during the COVID-19 pandemic. The literature review section outlines 3 main concepts: models of technology acceptance, SDOH (economic stability, access to education, access to health care, neighborhood and built environment, and social and community context), and how COVID-19 facilitated the adoption of telehealth.

### The History and Use of TAM

The concept of technology adoption became popular in the 1980s. It is imperative to establish accurate metrics for studying the attitudinal elements that mediate the link between information systems’ characteristics and their use. A theoretical model, such as the theory of reasoned action (TRA), has been used to assess technology use, acceptance, and adoption during that period [18]. The TRA was developed in 1967 by Martin Fishbein and Icek Ajzen [18] and is used to explain the relationship between attitudes and behaviors in human action. On the basis of the TRA, Fred D Davis developed the TAM [19]. The TAM depicts the acceptance and adoption of technology based on 3 users’ perceptions related to the use of technology. The first one is the perceived usefulness (PU) of technology, which is defined as “an individual’s perception of the extent to which the use of a given technology improves performance.” The second belief is perceived ease of use (PEOU), which is defined as “the degree to which a person believes that using a particular system is free of effort” [20]. The third belief is the IU, defined as “an individual’s intention or willingness to adopt and use technology” [20].

There are many variants of TAM, such as the original TAM, TAM2, and TAM3 [19]. TAM2 was developed to focus more on factors impacting PU, whereas TAM3 was designed to focus more on factors predicting PEOU [21]. We aimed to investigate the impacts of social determinants as external variables in the context of COVID-19. Therefore, we selected the original TAM as our framework because of its proven effectiveness in accurately predicting outcomes across a range of contexts. TAM2 and TAM3 introduced additional variables that are not necessary for our research [20].

### SDOH as External Variables of Technology Acceptance

The US Department of Health and Human Services defines SDOH as “the conditions in the environments where people are born, live, learn, work, play, worship, and age that affects a wide range of health, functioning, and quality-of-life outcomes and risks [22].” There are 5 main categories of SDOH: (1) economic stability, (2) access to education, (3) access to health care, (4) neighborhood and built environment, and (5) social and community context. These categories impact an individual’s and the community’s health status. Disparities in any category affect a measure called socioeconomic status (SES) [23]. Previous literature suggests that the lower the SES score, the poorer the health care outcomes, which would further lead to decreased life expectancy [19,24,25].

The first category, economic stability, includes subcategories such as employment, food security, and housing stability. The second category, education, primarily includes literacy levels and levels of education (lower than high school, middle or high school, college, and university graduates). Evidence suggests that higher levels of education correlate with increased life expectancy, largely because of enhanced access to health care services [25]. Low health literacy makes it difficult for patients to understand medical advice. Therefore, health care staff must provide medical information, keeping patients’ literacy and education levels in mind. Research also indicates that patients with health insurance are more likely to use health care services than patients without health insurance [26]. The fourth category, neighborhood and built environment, includes housing conditions, crime rates in the area, transportation, access to healthy food, and the quality of air and water. People living in deprived areas are more prone to stress than those living in better areas. The fifth category, social and community context, concerns where a person lives, learns, and works. The US Substance Abuse and Mental Health Services Administration emphasizes that access to technology and information plays a crucial role in making informed and health-conscious choices; therefore, technology should be regarded as a primary social determinant [27].

In addition, previous studies indicate that technological factors must be included as primary SDOH [28,29]. In our study, we have therefore included a sixth category, “technological factors,” because of the increased use of telehealth platforms during the COVID-19 pandemic. It could be argued that matters related to technology, ranging from availability to credibility, have significantly transformed communities nationwide throughout the pandemic, particularly affecting senior citizens and minority groups from underserved populations [30]. All of the above categories were connected and played an essential role in understanding health care access during the COVID-19 pandemic.

Health disparities are a long-standing issue in the US owing to the complex intersection of race, poverty, education quality and access, and the urban and rural divide [30]. Owing to the lack of access to services such as telehealth, the PEOU, PU, and IU technology among underserved populations are significantly less [30]. On the basis of the SDOH and the original TAM framework, we propose the following hypothesis:

- Hypothesis 1: SDOH factors, including economic stability, access to education, access to health care, neighborhood and built environment, and social and community context, will predict users’ PEOU, PU, and IU telehealth.

### COVID-19 as a Facilitating Condition of Telehealth Adoption

**Figure 1:**
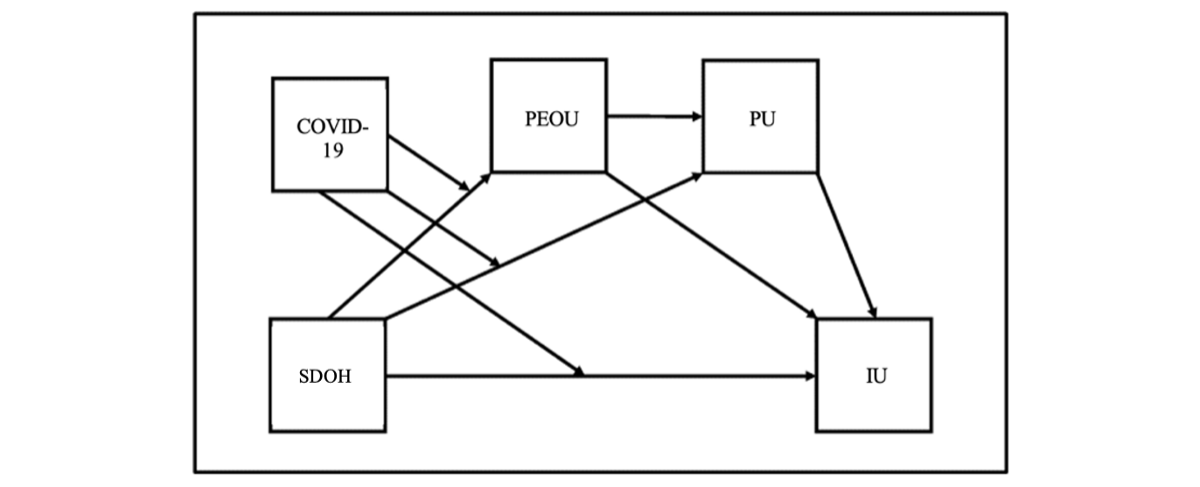
Conceptual model. IU: intention of use; PEOU: perceived ease of use; PU: perceived usefulness; SDOH: social determinants of health.

TAM also includes the effects of moderators. Research on the moderator effect began with the study by Adams et al [31] as early as the 1990s. TAM moderators are important because they provide a deeper understanding of the factors that influence individuals’ acceptance and use of technology [31,32]. TAM moderators enhance a model’s explanatory power by considering various contextual and individual factors that can influence the relationships within the model. Many studies have confirmed the significant influence of moderating factors in existing models of user technology acceptance [31,33]. Some moderators, such as experience, voluntariness, gender, and age, have been outlined in previous studies [33]. In this study, we sought to investigate the role of COVID-19 as both a predictor and moderator. Therefore, we propose 2 more hypotheses and a conceptual model outlining the relationships among all the variables (Figure 1):

- Hypothesis 2: having had COVID-19 before will predict their PEOU, PU, and IU related to telehealth.
- Hypothesis 3: having had COVID-19 before moderates the relationship between SDOH and PEOU, PU, and IU related to telehealth.

## Methods

### Participants

To test the study’s hypotheses, 215 participants were recruited through Amazon Mechanical Turk (MTurk) in May 2022 [34]. The survey was designed to be open and voluntary in nature. MTurk is a web-based crowdsourcing website owned and operated by Amazon. The platform allows participants to complete tasks for a small payment. Since 2010, numerous researchers have explored the viability of MTurk in recruiting participants for experiments [35-37]. The findings show that participants in MTurk are more demographically diverse than those in other web-based samples. Data for this study were collected from March 2022 to June 2022. Individuals >18 years of age were included in this study. Vulnerable groups such as children, pregnant women, individuals in nursing homes, and hospitalized individuals were excluded from this study. A total of 10 participants were excluded from the study because of missing data. Upon data cleaning, the sample size was reduced to 205 participants. All participants received compensation of US $1.

### Ethics Approval and Informed Consent

The institutional review board of the University of Pittsburgh approved this study (ID: STUDY21100192). We received a waiver for informed consent, as this study had no more than minimal risk. All information collected as part of the survey was stored in a secure password-protected device at the University of Pittsburgh. Only the research team (authors) had access to survey data.

### Survey Instrument

The web-based survey was conducted in accordance with the CHERRIES (Checklist for Reporting Results of Internet E-Surveys) checklist (Multimedia Appendix 1). The survey questionnaire comprised 4 sections: (1) SDOH, (2) telehealth and COVID-19, (3) TAM, and (4) demographics. All questions were obtained from validated questionnaires from previous research studies. The first part pertained to SDOH and was adopted from the study by Gold et al [38]. This section consists of 24 questions covering the following domains: economic stability, health care, education, neighborhood and build environment, social factors, and technological factors.

The second part consisted of 25 telehealth and COVID-19–related questions. The survey questions focused on whether participants had confirmed COVID-19 (tested positive) and their experiences of using telehealth services in general. The questionnaire items were averaged to obtain an overall scale score of 1 to 7. We included standard validated questions about the quality of services from the study by Imlach et al [39], with minor modifications.

The third section consisted of TAM-related questions adopted from the study by Kamal et al [33]. This section consisted of 22 questions. The questions in this section focused on 3 primary TAM constructs: PEOU, PU, and IU. Each construct included 2 or 3 dimensions. PEOU included questions related to telehealth and how the user interacts with the system (eg, interacting with telemedicine systems would be clear and understandable for me). PU included the usefulness of health care, the usefulness of access to health care, and the usefulness of daily routine (eg, using telemedicine would improve the quality of my health care). IU included more behavioral questions (eg, assuming that I was given a chance to access telemedicine, I intend to use telemedicine services). The response categories ranged from 1 (strongly disagree) to 7 (strongly agree). Analyzing these data through the lens of the TAM can provide insights into the factors affecting users’ acceptance of telehealth services. These insights can guide the improvement of telehealth platforms, user training, and communication strategies to enhance the adoption rates and overall user satisfaction.

The last section included 10 demographic questions on age, gender, ethnicity, education level, monthly income, and the presence of chronic conditions. The order of the sections presented in the questionnaire was SDOH, telehealth and COVID-19, technology acceptance variables, demographics, and control variables.

### Statistical Analysis

A total of 3 main analytical techniques were used in this study: descriptive analysis, ordinary least squares regression, and PROCESS moderation-mediation analysis. Data cleaning was conducted before data analysis, including consistency checks and the treatment of missing responses. Consistency checks are used to identify data that are out of range, are logically inconsistent, or have extreme values. Surveys with missing responses were excluded from the data set. All analyses were performed using SPSS Statistics (version 29; IBM Corp) [40].

### Descriptive Analysis

The first part of the data analysis used descriptive statistical analysis of variables by producing frequencies, means, ranges, and SDs to describe the sociodemographic details, whereas the clinical characteristics of patients were calculated for the usability and telehealth sections of the questionnaire.

### Descriptive Analysis of TAM Variables

TAM items were calculated and averaged based on the responses of 205 participants. Participants reported means on a scale from 1 (strongly disagree) to 7 (strongly agree) when they were asked about PEOU, PU, and IU telehealth services.

### Ordinary Least Squares Regression

The second part includes regressions to test the main effects of the SDOH and COVID-19. The analysis shows the standardized β (with 95% CIs) of TAM variables.

### PROCESS Moderated-Mediation Analysis

The third part used Hayes’ [41] PROCESS moderation-mediation analysis to determine the interaction and indirect effects. This study used the PROCESS model 85 with 5000 bootstrap samples and a 95% CI to test the proposed model.

## Results

### Descriptive Analysis

Table 1 summarizes the demographic characteristics of the participants. Approximately 55.1% (113/205) of the total participants were male. The age of respondents varied from 20 to 60 years, with a maximum frequency of respondents observed in the age groups of 30 and 40 years (85/205, 41.4%). The academic qualification of participants was observed primarily in the university category (129/205, 62.9%), followed by the postgraduate category (59/205, 28.7%). Approximately 94.6% (194/205) of the respondents had access to the internet, and 88.7% (181/205) had health insurance. Approximately 44.8% (92/205) of the respondents reported having tested positive for COVID-19. Among the respondents, 65.8% (135/205) used telehealth services. The population consisted of the following ethnic backgrounds: 85.3% (175/205) White; 5.8% (12/205) African American; 2.4% (5/205) Asian; 3.4% (7/205) Spanish, Hispanic, or Latino; and 1.9% (4/205) other. To assess the income and economic status of the participants, we asked them whether they were worried about losing their housing; 51.2% (105/205) of them reported being worried. In addition, we asked them whether they were unable to obtain utilities (heat, electricity, water, etc) when needed; 39% (80/205) of the participants were unable to do so. The study sample characteristics were in line with those found in other studies that examined MTurk demographic characteristics [42]. A specific study conducted on MTurk found that most respondents had an average age <50 years, were primarily White (approximately 75%), highly educated (attended university), and were currently employed (approximately 75%) [37].

**Table 1:** The demographic characteristics of the participants (N=205).

Variable			Frequency, n (%)
**Sex**			
	Male	113 (55.1)	
	Female	87 (42.4)	
	Unknown	5 (2.4)	
**Age (years)**			
	<20	1 (0.4)	
	20-30	41 (20.0)	
	30-40	85 (41.4)	
	40-50	45 (21.9)	
	50-60	19 (9.2)	
	>60	8 (3.9)	
	Unknown	6 (2.9)	
**Qualification**			
	High school or general educational development	17 (8.2)	
	University	129 (62.9)	
	Postgraduation	59 (28.7)	
	Less than high school	0 (0.0)	
**Do you have any access to internet facilities?**			
	Yes	194 (94.6)	
	No	11 (5.3)	
**Do you have a health insurance?**			
	Yes	181 (88.7)	
	No	23 (11.2)	
	Unknown	1 (0.4)	
**Have you ever been diagnosed with COVID-19?**			
	Yes	92 (44.8)	
	No	112 (54.6)	
	Unknown	1 (0.4)	
**Which of the following best describes your racial or ethnic background?**			
	White	175 (85.3)	
	Black or African American	12 (5.8)	
	American Indian or Alaska Native	2 (0.8)	
	Asian	5 (2.4)	
	Spanish, Hispanic, or Latino	7 (3.4)	
	Other	4 (1.9)	
**Did you use any specific telehealth apps or websites to get in touch with a physician virtually?**			
	Yes	135 (65.8)	
	No	70 (34.1)	
**Are you worried about losing your housing?**			
	Yes	105 (51.2)	
	No	100 (48.7)	
**Within the past 12 months, have you been unable to get utilities (heat, electricity, water, etc) when it was really needed?**			
	Yes	80 (39.0)	
	No	125 (60.1)	

### Descriptive Analysis of TAM Variables

Table 2 presents the descriptive statistics of the theoretical variables. The results showed that most respondents reported high scores on all 3 TAM variables (ranging from 4.73 to 5.14 out of 7), and the means of IU were the highest. Overall, these statistics suggest that the individuals who participated in the study had a moderately high perception of ease of use and usefulness of the technology, and a strong intention to use it in the future.

**Table 2:** Descriptive Analysis of technology acceptance model variables.

Construct (numbers of items; Cronbach α)	Response categories	Values, mean (SD)	Example
Perceived ease of use (2; .517)	From 1 to 7, 1=strongly disagree	4.730 (1.140)	Learning to use telemedicine would not be very difficult for me.
Perceived usefulness (3; .695)	From 1 to 7, 1=strongly disagree	5.053 (1.23)	Using telemedicine would improve the quality of my health care.
Intention to use (3; .613)	From 1 to 7, 1=strongly disagree	5.14 (1.06)	Whenever I would need remote medical care from professionals, I would gladly use telemedicine services.

### Ordinary Least Squares Regression

Table 3 displays TAM variables’ standardized β (with 95% CIs). This table presents the results of a series of ordinary least squares regression analyses with PEOU, PU, and IU as the dependent variables. The model includes 7 independent variables: 6 variables are social determinants (ie, economic stability, health care, education, neighborhood and built environment, social factors, and technological factors), and 1 variable is whether respondents have had COVID-19.

For PEOU, the results show that neighborhood and built environment are statistically significant (*P*=.01), whereas economic stability is marginally significant (*P*=.05). Other independent variables, including access to health care, education, social factors, and technological factors, were not statistically significant. Overall, these results suggest that neighborhood and built environment have the strongest positive impact on PEOU, whereas economic stability and COVID-19 are associated with higher PEOU. However, health, education, social, and technological factors did not appear to have a significant impact on PEOU.

For PU, the results show that the respondents’ access to health care and COVID-19 were statistically significant (*P*=.007 and *P*=.04, respectively), whereas the other independent variables were not statistically significant. The strongest predictor of PU is access to health care, with a β value of .193, indicating that a 1-SD increase in access to health care is associated with a 0.193 SD increase in PU. Similarly, COVID-19 was associated with higher PU scores. Overall, these results suggest that health-related factors and COVID-19 have a positive impact on PU, whereas economic stability, education, neighborhood and built environment, social factors, and technological factors do not appear to have a significant impact on PU. Regarding IU, the results show that none of the independent variables are statistically significant at the conventional significance level of .05.

Therefore, these results show that better access to health care services (including access to health insurance), safe neighborhoods, living conditions, and a stable economic status are all good predictors of PEOU and PU related to telehealth services. There was no significant difference in IU telehealth services.

**Table 3:** Ordinary least squares regression modeling of the impact of social determinants of health on the adoption of telehealth shows the standardized β of technology acceptance model variables (N=205).

Technology acceptance model parameters	Economic stability	Access to health care	Education	Neighborhood and built environment	Social factors	Technological factors	COVID-19
Perceived ease of use	0.140^a^	0.103	−0.049	0.191^b^	−0.001	−0.042	0.103^a^
Perceived usefulness	0.093	0.193^b^	−0.053	0.053	−0.018	−0.057	0.150
Intention to use	−0.02	0.043	0.02	−0.062	0.014	−0.120	0.07

^a^*P*<.05.

^b^*P*<.01.

### PROCESS Moderated-Mediation Analysis

Economic stability, and neighborhood and built environment are 2 predictors of PEOU. We ran 2 moderated mediation analyses using them as predictors. In both analyses, COVID-19 was the moderator, IU was the dependent variable, and PEOU and PU were the mediators (Figure 1).

Table S4 in Multimedia Appendix 2 shows the moderated mediation model for economic stability. For the PEOU variable, the independent variable “environment” (neighborhood and built environment) had a significant positive effect (β=.2361; *P*=.02) on the mediator variable. No other independent variables had a significant effect on the mediator variables. For the PU variable, the independent variables “economic stability” (β=.8074; *P*=.01) and “access to health care” (β=.6001; *P*=.007) had significant positive effects. No other independent variables had a significant effect on the outcome variables.

For the IU variable, the independent variables “PEOU” (β=.131; *P*=.02) and “PU” (β=.5707; *P*<.001) had significant positive effects, whereas “economic stability,” “COV,” “access to health care,” “education,” “social factors,” and “technological factors” did not have a significant effect on the dependent variable. This table suggests that the PEOU of technology is positively associated with its PU, which, in turn, is positively associated with users’ IU telehealth. Moreover, economic stability, COVID-19, access to health care, and PEOU have significant positive effects on PU. PEOU and PU have significant positive effects on users’ intentions to use technology.

Table S5 in Multimedia Appendix 3 shows the direct and indirect effects, and the difference between the conditional indirect effects in the proposed model. On the basis of the moderated mediation model results, there is an unconditional interaction effect of COVID-19 and economic stability on PU. The conditional effects showed that the interaction effects of COVID-19 and economic stability were significant among those who did not have COVID-19, but perceived the usefulness of telehealth. The unconditional interaction effect of COVID-19 and economic stability was not significant for PEOU and IU.

This moderated mediation path analysis also revealed 2 indirect effects of COVID-19 on IU. One was related to those who did not have COVID-19, whereas the other was related to those who had COVID-19. For those who did not have COVID-19 (COV=0), only economic stability had an indirect effect on IU through PEOU. For those with COVID-19 (COV=1), economic stability had an indirect effect on IU through both PEOU and PU. This finding indicates that the indirect effects of economic stability on IU were moderated by COVID-19, resulting in 2 different mediation relationships between people who have had COVID-19 and those who have not.

The second mediation-moderation analysis uses the variable neighborhood and built environment as predictors to test the direct and indirect effects and the difference between conditional indirect effects in the proposed model (see Tables S6 and S7 in Multimedia Appendices 4 and 5, respectively). First, the predictors (neighborhood and built environment) had a significant positive effect on PEOU (β=.3069; *P*=.03), indicating that a better neighborhood and built environment leads to an increased perception of ease of use. Similarly, in the PU model, PEOU (β=.4232; *P*<.001) and access to health care (β=.5373; *P*=.01) were significant predictors of PU. Finally, in the IU model, PEOU (β=.1252; *P*=.03) and PU (β=.5793; *P*<.001) were significant predictors of IU telehealth services.

**Figure 2:**
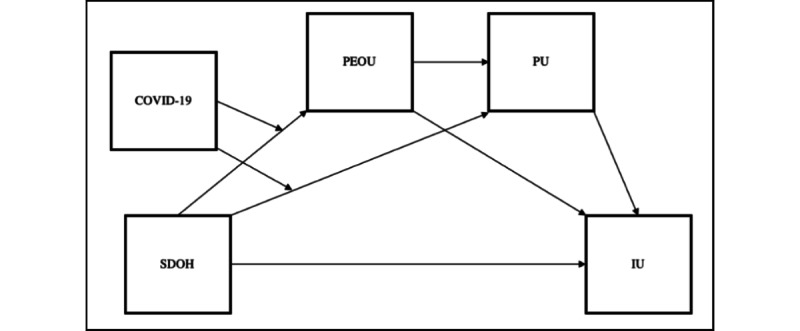
Revised conceptual model. IU: intention of use; PEOU: perceived ease of use; PU: perceived usefulness; SDOH: social determinants of health.

Regarding the interaction effects, there was an unconditional interaction effect of COVID-19 and the neighborhood and built environment on PU. The conditional effects showed that the interaction effects of COVID-19 and neighborhood and built environment were significant among those who had COVID-19. For PEOU and IU, the unconditional interaction effect of COVID-19 with neighborhood and built environment was not significant. The moderated mediation path analysis also revealed an indirect effect of COVID-19 on IU through both PEOU and PU, which showed that the environment could impact people’s IU telehealth services directly but also indirectly through PEOU and usefulness. Figure 2 shows the revised conceptual model.

## Discussion

### Summary of Key Findings

A total of 3 hypotheses are proposed in this study. The first hypothesis was that the SDOH predict telehealth’s PEOU, usefulness, and IU. The findings of the study suggest that 2 out of the 6 SDOH factors, namely economic stability, and neighborhood and built environment, were strong predictors of telehealth PU. Therefore, the first hypothesis is partially supported. Our findings are in line with the results found in the study by Chang et al [43], who show that SES or economic stability plays a crucial role in telehealth adoption. The study also emphasizes neighborhood and built environments, stating that unsafe neighborhoods make the population more susceptible to disasters and diseases, leading to a digital divide that shapes their inability to take full advantage of their telehealth capabilities [44].

The second hypothesis investigated whether COVID-19 is a predictor of telehealth, PEOU, PU, and IU. Our findings indicate that people diagnosed with COVID-19 were more likely to report a higher IU telehealth, which partially supports the second hypothesis. A possible explanation for this result is that the pandemic has increased the use of telehealth services in the country. Before the COVID-19 pandemic, telehealth was primarily used to address the lack of appropriate health care services in low-resource and rural settings [43]. With the surge in the number of COVID-19 cases worldwide, there has also been an advancement in technology that enables real-time care. With this rapid change in care delivery, most previous telehealth obstacles have almost disappeared. Telehealth was therefore adopted very quickly by hospitals, making health care more accessible to all in times of social distancing and other virus-related concerns. A total of 1 study revealed that patients largely appreciated and adopted telehealth as they did not have to leave their houses and fear the risk of infection [38].

The last hypothesis tested whether COVID-19 moderates the relationship between SDOH and telehealth. The results suggest an interaction effect of COVID-19 and SDOH factors (economic stability, and neighborhood and built environment) on the IU telehealth.

The study also tested the potential moderating role of COVID-19 on telehealth adoption (through TAM variables). The results suggest a conditional interaction effect of COVID-19 and telehealth on the intention to use it. In particular, COVID-19 led to PEOU and PU among those who used telehealth services during the pandemic. There were also 2 paths of conditional indirect effects on COVID-19, leading to IU through PEOU and PU. This finding suggests a moderated mediation relationship between COVID-19 and TAM variables. One explanation is that individuals who were not affected by COVID-19 possibly wanted to avoid hospitalization and, therefore, intended to use telehealth services. At the same time, those infected with COVID-19 perceived telehealth’s usefulness and intended to use it.

### Comparison With Existing Research

There is scant literature available on SDOH and their effects on telehealth adoption. Most of the available studies highlight the influence of factors such as race, ethnicity, and access to health care on the adoption of telehealth services [45-47]. Available evidence on ethnicity and race suggests that the majority of COVID-19 cases are recorded among racial minority groups [48]. For instance, even developed countries such as the United Kingdom and the US saw a high number of COVID-19 cases from racial minority groups [49]. The disproportion in the number of cases results from the health disparities and inequities experienced by minority communities. Recently, investigators have examined the effects of lower SES on health disparities; the findings identify and highlight that median household income is associated with a patient’s participation in telehealth.

A telehealth video-visit study was conducted by researchers at the Medical College of Wisconsin [48]. This study included 137,846 video visits involving 75,947 patients. The sociodemographic results of the study show that there were 81% White, 14% African American, 2% Asian, and less than 1% Alaska Native or American Indian. Approximately 23% of the study population were aged ≥65 years. Researchers primarily studied whether calls were successfully completed, and analyzed the reasons behind the drop-offs. Approximately 90% of the calls were successful in this study, whereas approximately 10% were unsuccessful. Upon further analysis, the researchers found that people with higher annual incomes were more likely to see successful visits. Some reasons were found to be that minority populations face broadband and technological obstacles. The study found that other sociodemographic factors, such as technology literacy and educational attainment, could largely influence the success of telehealth video visits.

Country- and state-wide lockdowns left families in isolation, during which they had to rely on internet searches and other digital means to obtain information about the COVID-19 pandemic [49]. Early studies indicate that although the internet provides a lot of information, people do not correctly use these resources [50,51]. In addition, these studies demonstrated that individuals with greater health literacy were able to differentiate between correct and incorrect COVID-19–related information. Several papers show that the educational level of an individual and digital literacy play a vital role in the adoption of telehealth services, in contrast to the results of this study [27,51,52]. In particular, racial minorities, older adults, and people with lower educational levels are not likely to engage in web-based patient portals despite having a stable internet connection [53-55]. Another interesting study compared whether an individual’s educational level or SES had a significant influence on telehealth use and adoption. The study found that a patient’s SES had a greater influence on telehealth adoption than educational or literacy levels.

Researchers have also studied other social determinant factors such as the presence or absence of health insurance and its influence on technology adoption [56]. A majority of these studies show that patients with health insurance are more likely to engage in virtual visits irrespective of their SES [56,57]. Of all the SDOH, limited literature is available on the influence of an individual’s neighborhood and built environment and its influence on technology adoption. The results of this study show that an individual’s neighborhood and built environment can influence telehealth adoption. However, more research is needed to completely understand the influence of SDOH on technology adoption.

### Research Implications, Limitations, and Future Directions

This pilot study shows that the SDOH influence technology adoption, especially in the context of the COVID-19 pandemic. The PEOU, PU, and IU telehealth among the general population were found to be high, and acceptance rates were much higher now than ever before. Further examining the data, we found that economic stability, access to health care, safe neighborhoods, and built environments play a vital role in adopting new technology, especially among underserved populations.

National and state governments must invest in educating people about health literacy in general and digital health literacy. The use and adoption of telehealth services were less common before the COVID-19 pandemic. Although the pandemic has increased the use of telehealth services, it is still bound by long-standing rules and regulations. Governments play a vital role in advancing the scope and impact of telehealth services. Therefore, robust policies and regulations must make these services more accessible to individuals from all backgrounds. Regarding theoretical contributions, this study connects 2 research frameworks to address unequal access to health technology. This study also adds empirical evidence to the telehealth research on patient adoption. Regarding practical implications, this study will give government agencies and health care organizations a better perspective on patients’ digital health use.

This study had several limitations. First, although factors associated with telehealth acceptance were included in this study, the actual behaviors of adopting telehealth were not analyzed. Second, our cross-sectional data could only provide a snapshot of participants’ responses at a particular point in time, highlighting the need for future longitudinal studies in the context of telehealth adoption. Third, this study did not consider other SDOH factors that may influence telehealth acceptance. Future studies should aim to incorporate these determinants to provide a more comprehensive understanding of barriers and facilitators to telehealth adoption. Fourth, although our pilot study leveraged MTurk for swift, cost-effective data collection, this might have led to sampling bias. We acknowledge the importance of a broader demographic representation and will pursue various recruitment strategies in future research. In addition, as this study focused solely on the US population, its findings may not be applicable to countries with distinct medical systems. Future studies should encompass more diverse international samples to enhance the findings’ applicability and generalizability of the findings.

Considering the broader picture, although there was an increase in the use of health technology during the COVID-19 pandemic, some studies have shown that people’s awareness of cybersecurity and data privacy also played an important role in adoption [58,59]. This study does not assess whether cybersecurity issues and data privacy are barriers to telehealth adoption. Future research should focus on incorporating these variables.

### Conclusions

We observed that disparities in the SDOH were an important indicator of telehealth adoption during the COVID-19 pandemic. Factors that influence adoption include gender, race, SES, level of education, and insurance type. Few studies have investigated the SDOH and telehealth adoption. Future studies should focus on the underlying factors of telehealth acceptance and use. This study adds to the literature that access to health care services, economic stability, neighborhood and built environment, and COVID-19 are primarily responsible for telehealth adoption among individuals. With the ever-increasing demand and implementation of telehealth services, governments and health care organizations across the globe must design better strategies to address barriers of technology adoption, especially among underserved populations.

## Appendix

Multimedia Appendix 1 CHERRIES (Checklist for Reporting Results of Internet E-Surveys).

Multimedia Appendix 2 Moderated mediation model for economic stability.

Multimedia Appendix 3 Direct and indirect effects for economic stability.

Multimedia Appendix 4 Moderated mediation model for neighborhood and built environment.

Multimedia Appendix 5 Direct and indirect effects for neighborhood and built environment.

## Abbreviations

IU: intention to use
PEOU: perceived ease of use
PU: perceived usefulness
SDOH: social determinants of health
SES: socioeconomic status
TAM: technology acceptance model
TRA: Theory of Reasoned Action

## References

[1] Koonin LM, Hoots B, Tsang CA, Leroy Z, Farris K, Jolly B, Antall P, McCabe B, Zelis CB, Tong I, Harris AM (2020). Trends in the use of telehealth during the emergence of the COVID-19 pandemic - United States, January-March 2020. MMWR Morb Mortal Wkly Rep.

[2] Mishra V (2020). Factors affecting the adoption of telemedicine during COVID-19. Indian J Public Health.

[3] Zughni LA, Gillespie AI, Hatcher JL, Rubin AD, Giliberto JP (2020). Telemedicine and the interdisciplinary clinic model: during the COVID-19 pandemic and beyond. Otolaryngol Head Neck Surg.

[4] Elawady A, Khalil A, Assaf O, Toure S, Cassidy C (2020). Telemedicine during COVID-19: a survey of health care professionals' perceptions. Monaldi Arch Chest Dis.

[5] Andrews E, Berghofer K, Long J, Prescott A, Caboral-Stevens M (2020). Satisfaction with the use of telehealth during COVID-19: an integrative review. Int J Nurs Stud Adv.

[6] Doraiswamy S, Abraham A, Mamtani R, Cheema S (2020). Use of telehealth during the COVID-19 pandemic: scoping review. J Med Internet Res.

[7] Bokolo Anthony Jnr (2020). Use of telemedicine and virtual care for remote treatment in response to COVID-19 pandemic. J Med Syst.

[8] Robbins T, Hudson S, Ray P, Sankar S, Patel K, Randeva H, Arvanitis TN (2020). COVID-19: a new digital dawn?. Digit Health.

[9] Wosik J, Fudim M, Cameron B, Gellad ZF, Cho A, Phinney D, Curtis S, Roman M, Poon EG, Ferranti J, Katz JN, Tcheng J (2020). Telehealth transformation: COVID-19 and the rise of virtual care. J Am Med Inform Assoc.

[10] Kannampallil T, Ma J (2020). Digital translucence: adapting telemedicine delivery post-COVID-19. Telemed J E Health.

[11] Zhang T, Mosier J, Subbian V (2021). Identifying barriers to and opportunities for telehealth implementation amidst the COVID-19 pandemic by using a human factors approach: a leap into the future of health care delivery?. JMIR Hum Factors.

[12] Tenforde AS, Borgstrom H, Polich G, Steere H, Davis IS, Cotton K, O'Donnell M, Silver JK (2020). Outpatient physical, occupational, and speech therapy synchronous telemedicine: a survey study of patient satisfaction with virtual visits during the COVID-19 pandemic. Am J Phys Med Rehabil.

[13] Gajarawala Shilpa N, Pelkowski Jessica N (2021). Telehealth benefits and barriers. J Nurse Pract.

[14] Levine S, Gupta R, Alkwatli K, Almoushref A, Cherian S, Jimenez DF, Cordero Baez GN, Hart A, Weinstock C (2022). Telehealth perceptions among us immigrant patients at an academic internal medicine practice: cross-sectional study. JMIR Hum Factors.

[15] Naik BN, Gupta R, Singh A, Soni SL, Puri GD (2020). Real-time smart patient monitoring and assessment amid COVID-19 pandemic - an alternative approach to remote monitoring. J Med Syst.

[16] Yao Rui, Zhang Wenli, Evans Richard, Cao Guang, Rui Tianqi, Shen Lining (2022). Inequities in health care services caused by the adoption of digital health technologies: scoping review. J Med Internet Res.

[17] Gray J, Partington A, Karnon J (2021). Access, use, and patient-reported experiences of emergency care during the COVID-19 pandemic: population-based survey. JMIR Hum Factors.

[18] Ajzen I, Fishbein M (1980). Understanding Attitudes and Predicting Social Behavior.

[19] Davis CS, Samuels EA (2021). Continuing increased access to buprenorphine in the United States via telemedicine after COVID-19. Int J Drug Policy.

[20] Davis FD (1989). Perceived usefulness, perceived ease of use, and user acceptance of information technology. MIS Q.

[21] Venkatesh V, Davis FD (2000). A theoretical extension of the technology acceptance model: four longitudinal field studies. Manage Sci.

[22] Healthy people 2030: social determinants of health. Office of Disease Prevention and Health Promotion.

[23] Singu S, Acharya A, Challagundla K, Byrareddy SN (2020). Impact of social determinants of health on the emerging COVID-19 pandemic in the United States. Front Public Health.

[24] Kruse C, Heinemann K (2022). Facilitators and barriers to the adoption of telemedicine during the first year of COVID-19: systematic review. J Med Internet Res.

[25] Gillie M, Ali D, Vadlamuri D, Carstarphen KJ (2022). Telehealth literacy as a social determinant of health: a novel screening tool to support vulnerable patient equity. J Alzheimers Dis Rep.

[26] Suran M (2022). Increased use of medicare telehealth during the pandemic. JAMA.

[27] Turcios Y (2023). Digital access: a super determinant of health. Substance Abuse and Mental Health Services Administration.

[28] Hauck K, Martin S, Smith P (2016). Priorities for action on the social determinants of health: empirical evidence on the strongest associations with life expectancy in 54 low-income countries, 1990-2012. Soc Sci Med.

[29] Sieck CJ, Sheon A, Ancker JS, Castek J, Callahan B, Siefer A (2021). Digital inclusion as a social determinant of health. NPJ Digit Med.

[30] Beech BM, Ford C, Thorpe RJ Jr, Bruce MA, Norris KC (2021). Poverty, racism, and the public health crisis in America. Front Public Health.

[31] Adams DA, Nelson RR, Todd PA (1992). Perceived usefulness, ease of use, and usage of information technology: a replication. MIS Q.

[32] Holtz Bree, Mitchell Katharine, Hirko Kelly, Ford Sabrina (2022). Using the technology acceptance model to characterize barriers and opportunities of telemedicine in rural populations: survey and interview study. JMIR Form Res.

[33] Kamal SA, Shafiq M, Kakria P (2020). Investigating acceptance of telemedicine services through an extended technology acceptance model (TAM). Technol Soc.

[34] Amazon Mechanical Turk.

[35] Hauser David J, Schwarz Norbert (2016). Attentive Turkers: MTurk participants perform better on online attention checks than do subject pool participants. Behav Res Methods.

[36] Mortensen K, Hughes Tl (2018). Comparing Amazon’s Mechanical Turk platform to conventional data collection methods in the health and medical research literature. J Gen Intern Med.

[37] Buhrmester Michael, Kwang Tracy, Gosling Samuel D (2011). Amazon's Mechanical Turk: a new source of inexpensive, yet high-quality, data?. Perspect Psychol Sci.

[38] Gold R, Bunce A, Cowburn S, Dambrun K, Dearing M, Middendorf M, Mossman N, Hollombe C, Mahr P, Melgar G, Davis J, Gottlieb L, Cottrell E (2018). Adoption of social determinants of health EHR tools by community health centers. Ann Fam Med.

[39] Imlach F, McKinlay E, Middleton L, Kennedy J, Pledger M, Russell L, Churchward M, Cumming J, McBride-Henry K (2020). Telehealth consultations in general practice during a pandemic lockdown: survey and interviews on patient experiences and preferences. BMC Fam Pract.

[40] SPSS software. IBM.

[41] Hayes AF (2012). PROCESS: a versatile computational tool for observed variable mediation, moderation, and conditional process modeling. ZBook.

[42] Walters K, Christakis DA, Wright DR (2018). Are Mechanical Turk worker samples representative of health status and health behaviors in the U.S.?. PLoS One.

[43] Chang JE, Lai AY, Gupta A, Nguyen AM, Berry CA, Shelley DR (2021). Rapid transition to telehealth and the digital divide: implications for primary care access and equity in a post-COVID era. Milbank Q.

[44] Hirko KA, Kerver JM, Ford S, Szafranski C, Beckett J, Kitchen C, Wendling AL (2020). Telehealth in response to the COVID-19 pandemic: implications for rural health disparities. J Am Med Inform Assoc.

[45] Mude W, Oguoma VM, Nyanhanda T, Mwanri L, Njue C (2021). Racial disparities in COVID-19 pandemic cases, hospitalisations, and deaths: a systematic review and meta-analysis. J Glob Health.

[46] Pareek M, Bangash MN, Pareek N, Pan D, Sze S, Minhas JS, Hanif W, Khunti K (2020). Ethnicity and COVID-19: an urgent public health research priority. Lancet.

[47] Khunti K, Singh AK, Pareek M, Hanif W (2020). Is ethnicity linked to incidence or outcomes of COVID-19?. BMJ.

[48] Crotty BH, Hyun N, Polovneff A, Dong Y, Decker MC, Mortensen N, Holt JM, Winn AN, Laud PW, Somai MM (2021). Analysis of clinician and patient factors and completion of telemedicine appointments using video. JAMA Netw Open.

[49] Holtgrave DR, Barranco MA, Tesoriero JM, Blog DS, Rosenberg ES (2020). Assessing racial and ethnic disparities using a COVID-19 outcomes continuum for New York State. Ann Epidemiol.

[50] Ng BP, Park C (2021). Accessibility of telehealth services during the COVID-19 pandemic: a cross-sectional survey of Medicare beneficiaries. Prev Chronic Dis.

[51] Yoon H, Jang Y, Vaughan PW, Garcia M (2020). Older adults' internet use for health information: digital divide by race/ethnicity and socioeconomic status. J Appl Gerontol.

[52] Sarkar U, Karter AJ, Liu JY, Adler NE, Nguyen R, Lopez A, Schillinger D (2011). Social disparities in internet patient portal use in diabetes: evidence that the digital divide extends beyond access. J Am Med Inform Assoc.

[53] Brown SH, Griffith ML, Kripalani S, Horst SN (2022). Association of health literacy and area deprivation with initiation and completion of telehealth visits in adult medicine clinics across a large health care system. JAMA Netw Open.

[54] El-Toukhy Sherine, Méndez Alejandra, Collins Shavonne, Pérez-Stable Eliseo J (2020). Barriers to patient portal access and use: evidence from the Health Information National Trends Survey. J Am Board Fam Med.

[55] Liang J, Aranda MP (2023). The use of telehealth among people living with dementia-caregiver dyads during the COVID-19 pandemic: scoping review. J Med Internet Res.

[56] Darrat I, Tam S, Boulis M, Williams AM (2021). Socioeconomic disparities in patient use of telehealth during the coronavirus disease 2019 surge. JAMA Otolaryngol Head Neck Surg.

[57] Braswell M, Wally MK, Kempton LB, Seymour RB, Hsu JR, Karunakar M, Afetse KE, Bailey G, Bosse M, Brownrigg M, Cuadra M, Dixon A, Girardi C, Grochowski E, Hysong A, Jolissaint J, Macknet D, Mayberry RM, Moody P, Peterson K, Phelps KD, Pollock H, Posey SL, Reid R, Roe K, Scannell B, Sims S, Stanley A, Wohler AD (2021). Age and socioeconomic status affect access to telemedicine at an urban level 1 trauma center. OTA Int.

[58] Glorin S (2021). A descriptive study on cybersecurity challenges of working from home during COVID-19 pandemic and a proposed 8 step WFH cyber-attack mitigation plan. Commun IBIMA.

[59] Sebastian G, George A, Jackson G (2023). Persuading patients using rhetoric to improve artificial intelligence adoption: experimental study. J Med Internet Res.

