# Supplementary material for: Social Determinants of Health and Patients’ Technology Acceptance of Telehealth During the COVID-19 Pandemic: Pilot Survey
Source: JMIR Hum Factors. 2023 Nov 7;10:e47982. doi: 10.2196/47982 (PMC10631497; doi:10.2196/47982)
Supplement: Multimedia Appendix 2 [file humanfactors_v10i1e47982_app2.docx]

| **Perceived Ease of Use** | | | | | | |
| --- | --- | --- | --- | --- | --- | --- |
| **Variables** | **β** | **SE** | **T** | ***P*** | **LLCI** | **ULCI** |
| Economic Stability | 0.1866 | 0.3791 | 0.4923 | 0.6231 | -0.5612 | 0.9344 |
| COV | -1.4 | 1.1371 | -1.2312 | 0.2198 | -3.6429 | 0.8429 |
| Int_1 | 0.8352 | 0.5712 | 1.4622 | 0.1453 | -0.2915 | 1.9618 |
| Access to Healthcare | 0.3199 | 0.2538 | 1.2604 | 0.2091 | -0.1807 | 0.8205 |
| Education | -0.1072 | 0.1985 | -0.5402 | 0.5897 | -0.4989 | 0.2844 |
| Environment | 0.2361 | 0.1011 | 2.3364 | 0.0205* | 0.0368 | 0.4355 |
| Social Factors | -0.0402 | 0.1857 | -0.2162 | 0.829 | -0.4065 | 0.3262 |
| Technological Factors | -0.2331 | 0.369 | -0.6317 | 0.5283 | -0.9611 | 0.4948 |
| **Perceived Usefulness** | | | | | | |
| **Variables** | **β** | **SE** | **T** | ***P*** | **LLCI** | **ULCI** |
| Economic Stability | 0.8074 | 0.3298 | 2.4481 | 0.0153* | 0.1568 | 1.458 |
| PEoU | 0.4509 | 0.0631 | 7.1495 | 0.000*** | 0.3265 | 0.5754 |
| COV | 3.3206 | 0.9925 | 3.3456 | 0.001*** | 1.3628 | 5.2784 |
| Int_1 | -1.5721 | 0.4994 | -3.1481 | 0.0019** | -2.5571 | -0.587 |
| Access to Healthcare | 0.6001 | 0.2216 | 2.7084 | 0.0074** | 0.163 | 1.0372 |
| Education | -0.1445 | 0.1727 | -0.8365 | 0.4039 | -0.4853 | 0.1962 |
| Environment | -0.0244 | 0.0891 | -0.2736 | 0.7847 | -0.2002 | 0.1514 |
| Social Factors | 0.0239 | 0.1615 | 0.1483 | 0.8823 | -0.2946 | 0.3425 |
| Technological Factors | -0.1309 | 0.3212 | -0.4075 | 0.6841 | -0.7645 | 0.5027 |
| **Intention to Use** | | | | | | |
| **Variables** | **β** | **SE** | **T** | ***P*** | **LLCI** | **ULCI** |
| Economic Stability | -0.264 | 0.2771 | -0.9527 | 0.342 | -0.8105 | 0.2826 |
| PEoU | 0.131 | 0.0588 | 2.2286 | 0.027* | 0.015 | 0.247 |
| PU | 0.5707 | 0.0602 | 9.4869 | 0.000*** | 0.452 | 0.6894 |
| COV | 0.3126 | 0.8448 | 0.37 | 0.7118 | -1.3539 | 1.9791 |
| Int_1 | -0.1996 | 0.4237 | -0.4711 | 0.6381 | -1.0354 | 0.6362 |
| Access to Healthcare | -0.2768 | 0.1868 | -1.4823 | 0.1399 | -0.6453 | 0.0916 |
| Education | 0.1498 | 0.1431 | 1.047 | 0.2965 | -0.1325 | 0.4322 |
| Environment | -0.1398 | 0.0737 | -1.8964 | 0.0594 | -0.2852 | 0.0056 |
| Social Factors | 0.0692 | 0.1336 | 0.518 | 0.6051 | -0.1943 | 0.3327 |
| Technological Factors | -0.3671 | 0.2657 | -1.3815 | 0.1688 | -0.8914 | 0.1571 |

*Note: N=205. *P<.05, **P<.01, ***P<.001. LLCI - low limit confidence interval; ULCI - upper limit confidence interval.*
