# Supplementary material for: Social Determinants of Health and Patients’ Technology Acceptance of Telehealth During the COVID-19 Pandemic: Pilot Survey
Source: JMIR Hum Factors. 2023 Nov 7;10:e47982. doi: 10.2196/47982 (PMC10631497; doi:10.2196/47982)
Supplement: Multimedia Appendix 3 [file humanfactors_v10i1e47982_app3.docx]

| **Unconditional Interaction** | | | | | |
| --- | --- | --- | --- | --- | --- |
| **Perceived ease of use** | R2-chng | F | df1 | df2 | *P* |
| EcoStab   x    COV | .0102 | 2.1380 | 1.0000 | 190.0000 | .1453 |
| **Unconditional Interaction** | | | | | |
| **Perceived usefulness** | R2-chng | F | df1 | df2 | *P* |
| EcoStab    x    COV | .0373 | 9.9106 | 1.0000 | 189.0000 | .0019** |
| **Conditional effects** | | | | | |
| **Perceived usefulness** | Effect | se | LLCI | ULCI |  |
| .0000 | .8074 | .3298 | .1568 | 1.4580 |  |
| 1.0000 | -.7647 | .3799 | -1.5140 | -.0153 |  |
| **Unconditional Interaction** | | | | | |
| **Intention to use** | R2-chng | F | df1 | df2 | *P* |
| EcoStab   x    COV | .0006 | .2219 | 1.0000 | 188.0000 | .6381 |
| **Direct and Indirect Effects** | | | | | |
| **Conditional direct effects of X on Y** | | | | | |
| COV | Effect | se | LLCI | ULCI |  |
| 0 | -.2640 | .2771 | -.8105 | .2826 |  |
| 1 | -.4636 | .3175 | -1.0899 | .1628 |  |
| **Conditional indirect effects of X on Y:  Economic Stability      ->    PEoU        ->    IU** | | | | | |
| COV | Effect | BootSE | BootLLCI | BootULCI |  |
| 0 | .0245 | .0665 | -.0891 | .1912 |  |
| 1 | .1339 | .0918 | -.0029 | .3504 |  |
| **Index of moderated mediation (difference between conditional indirect effects)** | | | | | |
| Index | BootSE | BootLLCI | BootULCI |  |  |
| .1094 | .1004 | -.0523 | .3467 |  |  |
| **Indirect Effect:  Economic Stability    ->    PU          ->    IU** | | | | | |
| COV | Effect | BootSE | BootLLCI | BootULCI |  |
| 0 | .4608 | .1829 | .0899 | .8155 |  |
| 1 | -.4364 | .2219 | -.8610 | .0145 |  |
| **Index of moderated mediation (difference between conditional indirect effects)** | | | | | |
| Index | BootSE | BootLLCI | BootULCI |  |  |
| -.8972 | .2984 | -1.4697 | -.3066 |  |  |
| **Indirect Effect:  Economic Stability     ->    PEoU        ->    PU          ->    IU** | | | | | |
| COV | Effect | BootSE | BootLLCI | BootULCI |  |
| 0 | .0480 | .1099 | -.1599 | .2733 |  |
| 1 | .2630 | .1201 | .0541 | .5260 |  |
| **Index of moderated mediation (difference between conditional indirect effects)** | | | | | |
| Index | BootSE | BootLLCI | BootULCI |  |  |
| 0.2149 | .1586 | -.0770 | .5499 |  |  |
