# Supplementary material for: Social Determinants of Health and Patients’ Technology Acceptance of Telehealth During the COVID-19 Pandemic: Pilot Survey
Source: JMIR Hum Factors. 2023 Nov 7;10:e47982. doi: 10.2196/47982 (PMC10631497; doi:10.2196/47982)
Supplement: Multimedia Appendix 4 [file humanfactors_v10i1e47982_app4.docx]

| **Perceived Ease of Use** | | | | | | |
| --- | --- | --- | --- | --- | --- | --- |
| **Variables** | **β** | **SE** | **T** | ***P*** | **LLCI** | **ULCI** |
| Constant | 2.3473 | 1.1175 | 2.1005 | .0370 | .1430 | 4.5516 |
| Environment | .3069 | .1416 | 2.1667 | .0315 | .0275 | .5862 |
| COV | .5944 | .5866 | 1.0133 | .3122 | -.5627 | 1.7515 |
| Int_1 | -.1186 | .1896 | -.6255 | .5324 | -.4927 | .2555 |
| Economic Stability | .5509 | .2871 | 1.9186 | .0565 | -.0155 | 1.1172 |
| Access to Healthcare | .3708 | .2530 | 1.4653 | .1445 | -.1284 | .8699 |
| Education | -.1487 | .1981 | -.7504 | .4539 | -.5395 | .2421 |
| Social Factors | -.0004 | .1858 | -.0022 | .9982 | -.3670 | .3662 |
| Technological Factors | -.1569 | .3772 | -.4160 | .6779 | -.9010 | .5872 |
| **Perceived Usefulness** | | | | | | |
| **Variables** | **β** | **SE** | **T** | ***P*** | **LLCI** | **ULCI** |
| Constant | 1.3621 | .9892 | 1.3770 | .1701 | -.5891 | 3.3133 |
| Environment | .1700 | .1255 | 1.3553 | .1769 | -.0774 | .4175 |
| PEoU | .4232 | .0635 | 6.6664 | .0000 | .2980 | .5484 |
| COV | 1.3915 | .5147 | 2.7034 | .0075* | .3762 | 2.4068 |
| Int_1 | -.3916 | .1661 | -2.3574 | .0194 | -.7193 | -.0639 |
| Economic Stability | .1373 | .2537 | .5413 | .5889 | -.3631 | .6377 |
| Access to Healthcare | .5373 | .2227 | 2.4131 | .0168* | .0981 | .9766 |
| Education | -.0995 | .1736 | -.5732 | .5672 | -.4420 | .2430 |
| Social Factors | -.0070 | .1626 | -.0430 | .9658 | -.3278 | .3138 |
| Technological Factors | -.0439 | .3303 | -.1329 | .8944 | -.6953 | .6076 |
| **Intention to Use** | | | | | | |
| **Variables** | **β** | **SE** | **T** | ***P*** | **LLCI** | **ULCI** |
| Constant | 3.3846 | .8135 | 4.1607 | .0000 | 1.7799 | 4.9894 |
| Environment | -.1575 | .1032 | -1.5264 | .1286 | -.3610 | .0460 |
| PEoU | .1252 | .0577 | 2.1687 | .0314 | .0113 | .2391 |
| PU | 5793 | .0595 | 9.7329 | .0000 | .4619 | .6967 |
| COV | -.1745 | .4293 | -.4065 | .6849 | - 1.0213 | .6723 |
| Int_1 | .0314 | .1379 | .2275 | .8203 | -.2407 | .3035 |
| Economic Stability | -.3510 | .2077 | -1.6896 | .0928 | -.7608 | .0588 |
| Access to Healthcare | -.2928 | .1850 | -1.5828 | .1151 | -.6578 | .0721 |
| Education | .1603 | .1422 | 1.1271 | .2611 | -.1202 | .4408 |
| Social Factors | .0598 | .1331 | .4490 | .6540 | -.2028 | .3223 |
| Technological Factors | -.3853 | .2703 | -1.4256 | .1557 | -.9184 | .1479 |
