# Supplementary material for: Social Determinants of Health and Patients’ Technology Acceptance of Telehealth During the COVID-19 Pandemic: Pilot Survey
Source: JMIR Hum Factors. 2023 Nov 7;10:e47982. doi: 10.2196/47982 (PMC10631497; doi:10.2196/47982)
Supplement: Multimedia Appendix 5 [file humanfactors_v10i1e47982_app5.docx]

| **Unconditional Interaction** | | | | | |
| --- | --- | --- | --- | --- | --- |
| **Perceived ease of use** | R2-chng | F | df1 | df2 | *P* |
| Environment x COV | 0.0019 | .3913 | 1.0000 | 190.0000 | .5324 |
| **Unconditional Interaction** | | | | | |
| **Perceived usefulness** | R2-chng | F | df1 | df2 | *P* |
| Environment x  COV | .0214 | 5.5571 | 1.0000 | 189.0000 | .0194 |
| **Conditional effects** | | | | | |
| **Perceived usefulness** | Effect | se | LLCI | ULCI |  |
| 0 | .1700 | .1255 | -.0774 | .4175 |  |
| 1 | -.2216 | .1196 | -.4575 | .0144 |  |
| **Unconditional Interaction** | | | | | |
| **Intention to use** | R2-chng | F | df1 | df2 | *P* |
| Environment x  COV | .0001 | .0518 | 1.0000 | 188.0000 | .8203 |
| **Direct and Indirect Effects** | | | | | |
| **Conditional direct effects of X on Y** | | | | | |
| COV | Effect | se | LLCI | ULCI |  |
| 0 | -.1575 | .1032 | -.3610 | .0460 |  |
| 1 | -.1261 | .0988 | -.3209 | .0687 |  |
| **Conditional indirect effects of X on Y:  Environment**  **->    PEoU        ->    IU** | | | | | |
| COV | Effect | BootSE | BootLLCI | BootULCI |  |
| 0 | .0384 | .0255 | -.0024 | .0969 |  |
| 1 | .0236 | .0245 | -.0114 | .0830 |  |
| **Index of moderated mediation (difference between conditional indirect effects)** | | | | | |
| Index | BootSE | BootLLCI | BootULCI |  |  |
| -.0149 | .0270 | -.0708 | .0381 |  |  |
| **Indirect Effect:  Environment**  **->    PU          ->    IU** | | | | | |
| COV | Effect | BootSE | BootLLCI | BootULCI |  |
| 0 | .0985 | .0660 | -.0416 | .2220 |  |
| 1 | -.1284 | .0798 | -.2936 | .0240 |  |
| **Index of moderated mediation (difference between conditional indirect effects)** | | | | | |
| Index | BootSE | BootLLCI | BootULCI |  |  |
| -.2269 | .1005 | -.4267 | -.0214 |  |  |
| **Indirect Effect:  Environment**  **->    PEoU        ->    PU          ->    IU** | | | | | |
| COV | Effect | BootSE | BootLLCI | BootULCI |  |
| 0 | .0752 | .0342 | .0121 | .1480 |  |
| 1 | .0462 | .0364 | -.0185 | .1262 |  |
| **Index of moderated mediation (difference between conditional indirect effects)** | | | | | |
| Index | BootSE | BootLLCI | BootULCI |  |  |
| -.0291 | .0460 | -.1162 | .0664 |  |  |
